# Study protocol for a randomized trial of a supportive care mobile application to improve symptoms, coping, and quality of life in patients with advanced non-small cell lung cancer

**DOI:** 10.3389/fpsyg.2023.1184482

**Published:** 2023-06-22

**Authors:** Lauren P. Waldman, Joely A. Centracchio, Jamie M. Jacobs, Laura A. Petrillo, Areej R. El-Jawahri, Jennifer S. Temel, Joseph A. Greer

**Affiliations:** Massachusetts General Hospital, Boston, MA, United States

**Keywords:** palliative care, supportive care, coping, quality of life, symptom management, non-small cell lung cancer, mobile application (app)

## Abstract

Patients with advanced non-small cell lung cancer (NSCLC) often experience burdensome symptoms, emotional distress, and poor quality of life (QOL). While national guidelines recommend early palliative care to address these supportive care needs, most patients with advanced NSCLC lack access to such comprehensive care. Our aim in the current study is to test a novel model of palliative care delivery and use of innovative technology to evaluate the feasibility, acceptability, and preliminary efficacy of a supportive care mobile application (app) for improving symptom management and adaptive coping in patients with advanced NSCLC. We will enroll 120 patients with unresectable Stage III or IV NSCLC diagnosed within the past 12 weeks receiving care with palliative intent at a major academic comprehensive cancer center and its community affiliates. The study will take place in two phases, the first of which will be dedicated to adapting an evidence-based, early palliative care treatment guide and prior supportive care mobile app intervention to address the specific symptom management and coping needs of patients with advanced NSCLC. The second phase of the study will be a two-group, randomized controlled trial. Study patients will complete baseline self-report measures of symptoms, mood, coping skills, and QOL, after which they will be randomized to receive either the mobile app intervention combined with usual oncology care or usual oncology care alone. Intervention patients will use a tablet computer to self-administer the mobile app, which consists of six modules that teach evidence-based skills for managing burdensome symptoms and coping effectively with advanced cancer and its treatment. At 12 weeks follow up, patients in both groups will repeat the same self-report measures. We will use descriptive statistics to determine feasibility metrics of enrollment and retention rates. For secondary self-report measures, we will use linear regression controlling for baseline values. The results of the present study will contribute to a growing body of evidence regarding the supportive care needs of patients with advanced cancer and will have implications for how best to use innovative technology to widely disseminate comprehensive supportive care services to all patients who may benefit.

**Clinical Trial Registration:** [www.ClinicalTrials.gov], identifier[NCT04629300].

## 1. Introduction

Despite remarkable breakthroughs in cancer therapeutics prolonging survival, many patients with advanced lung cancer continue to experience high symptom burden, psychological distress, and poor quality of life (QOL; Morrison et al., 2017). To address these unmet supportive care needs, our multidisciplinary research team of clinician scientists who specialize in thoracic oncology, palliative care, and clinical psychology has been investigating the effects of integrating palliative care in the outpatient oncology setting for patients with advanced lung cancer. Multiple randomized trials and follow-up studies have demonstrated the positive effects of this integrated model of care on patient QOL, mood symptoms, prognostic awareness, and quality of end-of-life care (Temel et al., 2010, 2011, 2017; Greer et al., 2012). Moreover, the beneficial effects of the early, integrated palliative care intervention on QOL and mood symptoms appear to be accounted for by improvements in patients’ use of adaptive coping skills (Greer et al., 2018). Descriptive analyses of clinician documentation regarding topics addressed during outpatient palliative care visits from prior trials indeed confirm that the top two primary foci were symptom management and helping patients cope effectively with cancer and its treatment (Hoerger et al., 2018).

However, the field of supportive oncology has failed to translate evidence-based interventions, such as early, integrated palliative care, from randomized controlled trials into disseminated clinical services that are widely accessible and tailored to the unique experiences of patients and caregivers. Only a minority of patients with cancer and their caregivers receive or have access to supportive care, including early, integrated palliative care services (Sullivan et al., 1702; Dionne-Odom et al., 2018). Moreover, a retrospective cohort study using the SEER-Medicare linked database revealed that, from 2000 to 2009, rates of palliative care consultations for patients with advanced cancer only increased from 3.0 to 12.9% despite guidelines that recommend palliative care for all patients (Roeland et al., 2016; Ferrell et al., 2017). Most of these consultations occurred in the inpatient setting (83%) and during the final month of life (77%; Roeland et al., 2016). A major barrier to the dissemination of early, integrated palliative care is the lack of specialty trained clinicians available to provide this high quality, evidence-based intervention (Lupu, 2010; Lupu et al., 2018).

Mobile applications (apps) offer a useful approach for enhancing the accessibility and scalability of interventions addressing the supportive care needs of patients who cannot access health care clinicians with necessary expertise. Once patients receive appropriate instructions on their use, mobile app interventions are highly acceptable to patients regardless of their age, health literacy, or computer experience (Vernon, 2010; Basch et al., 2016). Moreover, the growing use of mobile apps across various age, ethnic, and socioeconomic groups has started to reduce the “digital divide” observed in prior studies of interventions administered through the internet on desktop computers (Carroll et al., 2017). Mobile behavioral interventions have shown promising efficacy in addressing a variety of health problems including depression, anxiety, substance use disorder, and insomnia in the general population and in patients with psychiatric conditions (Bewick et al., 2008; Andersson and Cuijpers, 2009; Cuijpers et al., 2009; Ritterband et al., 2009; Vernon, 2010; Mohr et al., 2013; Cuijpers et al., 2014). While some studies of behavioral web-based and mobile interventions have demonstrated low adherence, mobile apps that incorporate features to enhance patients’ engagement including the use of tailored logic, educational games (i.e., gamification), adaptive skill-building, and a record of personal progress through the app content, have shown greater than 85% usability and retention rates (Payne et al., 2015). To overcome the challenges due to limited access to supportive care for patients with advanced cancer, our multidisciplinary research team has begun to develop and test the use of mobile apps. These apps include interventions based on CBT, psychoeducation, and behavioral self-management aimed at improving outcomes such as anxiety, symptom burden, and adherence to oral chemotherapy. The results from these trials showed that participants with worse baseline distress or adherence problems benefited most from the mobile app interventions (Fishbein et al., 2017; Greer et al., 2019, 2020).

Thus, the primary aim for the present study is to develop a novel supportive care mobile app by adapting our team’s existing mobile app interventions to address the specific symptom management and coping needs of patients with advanced NSCLC. We will examine the feasibility, acceptability, and preliminary efficacy of the mobile app to improve symptoms, coping skills, and QOL in patients with advanced NSCLC. We also aim to understand patients’ general perceptions of the mobile app and their overall satisfaction with the intervention by conducting qualitative exit interviews at the end of the study.

## 2. Methods and analysis

### 2.1. Design

The proposed study includes two phases for (a) mobile app development and refinement and (b) pilot efficacy testing. The first preliminary phase was dedicated to adapting our evidence-based, early palliative care treatment guide and prior supportive care mobile app interventions to address the specific symptom management and coping needs of patients with advanced NSCLC (Jacobsen et al., 2021). Our team’s prior research on mobile app interventions, specifically adapted to other populations of patients with advanced cancer, focused largely on addressing physical symptoms and emotional well-being. While patients with NSCLC do similarly experience obstacles across these domains, we have also found that they face challenges regarding their social, functional, and existential well-being. Given their complex and broad care needs across multiple domains, our research team comprised of clinician scientists with expertise in thoracic oncology, palliative care, and clinical psychology specifically designed and tailored our app to address each of these domains. We collaborated closely with a mobile app development and design company to create, refine, and finalize the supportive care mobile app, called “THRIVE.” We met with this technology partner weekly for approximately 1 year and communicated regularly online over Basecamp to review and refine scripted content for each learning module as well as illustrative graphics and interactive exercises to enhance patient engagement with learning the coping skills and symptom management strategies. Each module was constructed based on evidence-based psychoeducation, CBT, and acceptance and commitment therapy (ACT) principles. We completed the development and programming of all the THRIVE mobile app modules, and we conducted extensive user testing to identify and resolve any bugs, glitches, graphic errors, or other performance issues. Table 1 outlines the domains of the supportive care modules and corresponding skills-based interventions that the app includes. This phase was completed by September 2021.

**Table 1:** THRIVE mobile app components.

Module	Content
Introduction	Learn about the features, functionality, and components of the THRIVE app Review ‘roadmap’ of key medical milestones from diagnosis through phases of treatment for advanced non-small cell lung cancer Meet clinician guide for the app and watch patient personal narrative video
Physical Domain (“Health”)	Identify and evaluate symptoms Learn to communicate effectively with oncology team about symptoms Apply helpful self-management strategies for coping with symptoms
Social Domain (“Relationships”)	Clarify types of social support needs related to coping with cancer Identify those who can help meet those needs Learn effective communication skills to ask others for help
Emotional Domain (“Emotions”)	Identify the range of cancer-related worries and emotions Practice skills for coping with uncertainty about the future Learn to overcome avoidance behaviors and to foster positive emotions
Functional Domain (“Lifestyle”)	Identify patterns in energy and stamina during treatment Practice skills for managing energy fluctuations and improving self-care Learn to prioritize and pace activities as needed to achieve valued life goals
Existential Domain (“Reflection”)	Reflect on life history, relationships, and goals Participate in activities that increase meaning and purpose in life despite cancer Learn to talk with the oncology team about how to prepare for the future

The second phase consists of a multi-site pilot randomized controlled trial in a sample of 120 patients with advanced NSCLC to assess the feasibility, acceptability, and preliminary efficacy of the THRIVE mobile app intervention for improving patient-reported symptoms, coping, and QOL compared to usual oncology care. Enrolled patients are randomized in 1:1 fashion and stratified by study site (i.e., Massachusetts General Hospital [MGH] Cancer Center, Dana-Farber Cancer Institute, and four community affiliates) to receive either the THRIVE mobile app combined with usual oncology care or usual oncology care alone. After enrollment, participants complete baseline self-report measures regarding their symptoms, mood, coping behaviors, and QOL, which they repeat at 12 weeks post baseline. We also conduct qualitative exit interviews with up to 30 patients who are randomized to the mobile app intervention to understand and assess patients’ experiences using the THRIVE app.

### 2.2. Selection/treatment of subjects

#### 2.2.1. Screening for eligibility

Trained RAs screen the oncology clinic schedules at the study sites to identify potentially eligible patients. The target sample is 120 patients with newly diagnosed advanced NSCLC. Once a patient is deemed eligible in the screening process and approval is obtained from their treating oncologist, the RA approaches the patient in a private clinic setting at their next oncology visit or *via* telephone to describe the study, confirm eligibility, and complete informed consent procedures. The research team also may provide study information to eligible patients *via* a secure patient portal, using templated language. Specifically, patients receive a summary of the study and are encouraged to contact the research team if interested in learning more about participating in the study.

Participant inclusion/exclusion criteria are as follows:

##### 2.2.1.1. Inclusion criteria

- Age ≥ 18 years.
- Diagnosed with unresectable Stage III or IV NSCLC in the past 12 weeks and receiving care with palliative intent.
- Eastern Cooperative Oncology Group (ECOG) Performance Status = 0–2 (with 0 indicating that the patient is asymptomatic, 1 that the patient is symptomatic but fully ambulatory, and 2 that the patient is symptomatic but in bed < 50% of the day; Cella et al., 2022).
- Plan to receive oncology care at one of the participating study sites.
- Ability to read and respond to questions in English.

##### 2.2.1.2. Exclusion criteria

- Significant uncontrolled psychiatric disorder (e.g., psychotic disorder, bipolar disorder, major depression) or other co-morbid disease (e.g., dementia, cognitive impairment), which the treating oncology clinician reports would prohibit the ability of the patient to participate in study procedures.

#### 2.2.2. Registration and randomization

Enrolled patients are registered in the Clinical Trials Management System, OnCore, as required by Dana-Farber/Harvard Cancer Center standard operating procedures. Once the patient has been registered, a member from the MGH Cancer Outcomes Research and Education Program (independent from the study staff) performs randomization procedures using a predetermined computer-generated randomization schema developed and stored independently from the study team with 1:1 allocation, stratified by study site. Given the interventional nature of the study, neither participants nor study staff are blinded to group assignment.

### 2.3. Interventional methods

Upon study enrollment, participants complete baseline self-report measures, after which they are randomly assigned to either the THRIVE mobile app (intervention) with usual oncology care group or the usual oncology care alone group (control).

#### 2.3.1. Intervention group

For patients randomized to the THRIVE intervention, the RA provides them with a study-issued tablet computer from which the participants access the mobile app or assists the patient in downloading the app onto a suitable device already owned by the participant. The mobile app is optimized for the iOS operating system. The RA provides intervention patients with a comprehensive tutorial and detailed instructions regarding how to use the app and is available throughout the study to provide technological support if needed. Intervention patients also receive a handout with application download instructions, an informational video sent securely *via* email, and a user guide. Patients complete the mobile app intervention modules at their desired pace over approximately 10 weeks. The app prompts patients with reminders to complete their modules and utilizes incentives to enhance patient engagement. Table 1 details the components of the THRIVE mobile app modules. The mobile app electronically collects data on intervention fidelity (e.g., the number of completed modules, proportion of each module completed, time spent on each module, etc.).

Finally, we conduct qualitative interviews with up to 30 patients who are randomized to the intervention group to learn about their experiences with the THRIVE mobile app. We purposively sample patients to ensure adequate representation based on age and gender. The semi-structured interviews are conducted after the 12-week post assessment time point.

#### 2.3.2. Usual oncology care group

Patients assigned to the usual oncology care group receive standard cancer care without the THRIVE mobile app. As part of usual care, we query the electronic health record to track and record participant use of any supportive care services, such as social work, psychology, psychiatry, and palliative care. While we expect the referral and use of these services to be low and balanced between study groups, we will include these variables as covariates in the outcome analyses as needed.

#### 2.3.3. Self-report measures

The study staff administers the following validated, self-report questionnaires at baseline prior to randomization and at approximately 12 weeks (+/− 2 weeks) after baseline. Participants are given a four-week time frame surrounding the 12-week time point to complete the measures. This window, which has consistently been used by our research group, allows for greater flexibility for participants to complete these measures given their degree of medical morbidity. These measures can be administered *via* paper hard-copy or electronically using Research Electronic Data Capture (REDCap), a secure, online HIPAA-compliant survey tool (Harris et al., 2009). The RA asks study participants to complete questionnaires in-person during clinic visits, *via* email, or over the telephone if necessary.

- Socio-demographic characteristics: Participants self-report their gender, race, ethnicity, marital status, education level, employment status, income, and frequency of technology use on a demographic questionnaire.
- Quality of life: Functional Assessment of Cancer Therapy-Lung (FACT-L; Cella et al., 1995). The FACT-L consists of four subscales assessing physical, social, emotional, and functional wellbeing during the past week. The instrument also contains the Lung Cancer Subscale (LCS), which evaluates specific symptoms related to lung cancer. Scores range from 0 to 5 on each item and the total scale score ranges from 0 to 136 with higher scores indicating better QOL.
- Symptoms: MD Anderson Symptom Inventory (MDASI; Cleeland et al., 2000). The MDASI is a 19-item survey, which consists of two subscales to assess cancer-related symptom severity and interference with daily activities on a 0–10 scale, with higher scores indicating worse symptoms.
- Mood: Hospital Anxiety and Depression Scale (HADS; Zigmond and Snaith, 1983). The 14-item HADS includes two subscales that measure anxiety and depression symptoms in the past week. Scores range from 0 to 21 on each subscale, and a threshold of > 7 on either subscale indicates clinically significant anxiety or depression symptoms.
- Coping strategies: Brief COPE Questionnaire; (Carver et al., 1989; Carver, 1997). The Brief COPE consists of 28 items assessing diverse coping methods on 14 subscales (e.g., active coping, use of emotional support, positive reframing, acceptance, behavioral disengagement, denial, and self-blame). Each item is scored on a 4-point Likert scale, with each subscale ranging from 0 to 8. Higher scores indicate higher use of that coping strategy. To reduce participant burden, we modified the questionnaire to consist of 8 subscales (Hagan et al., 2017).
- Supportive care service use: The Supportive Care Service Use Questionnaire (SCSUQ) includes two items that measure a patient’s use of mental health services, integrative medicine interventions, palliative care, and spiritual support over the past month.
- Mobile App Usability (intervention group only at week 12): The System Usability Scale (SUS; Bangor et al., 2008). The 10-item SUS evaluates patients’ subjective assessment of a product’s usability and acceptability.
- Perceptions and satisfaction with the mobile app (up to 30 intervention participants): Using a semi-structured qualitative interview guide, we assess patients’ experiences and satisfaction with the mobile app intervention.

#### 2.3.4. Electronic health record review

Study staff collects data from the electronic health record regarding patient age, cancer diagnosis, ECOG performance status, cancer therapy information, emergency department visits, hospitalizations, hospice referral, and use of supportive care services.

### 2.4. Data analysis

We will first report baseline characteristics of all study participants using descriptive statistics. The primary endpoint of the proposed study is feasibility. To assess intervention feasibility, we will calculate rates of patient enrollment, retention, intervention completion, and attrition while documenting reasons for withdrawal or loss to follow-up. The proposed intervention app will be deemed feasible if at least 65% of patients with advanced NSCLC who are approached to enroll in the study consent to participate and at least 70% complete the majority of study procedures as assigned (i.e., at least four of the six mobile app modules as well as baseline and 12-week outcome measures). These feasibility and intervention module completion thresholds are commonly used in behavioral intervention studies (Steinhauser et al., 2006; Siddiqi et al., 2008; Bowen et al., 2009). We will also use descriptive statistics to examine the acceptability and usability of the mobile app based on the System Usability Scale (Bangor et al., 2008).

To evaluate secondary outcomes, we will compare participant QOL (FACT-L; primary), symptoms (MDASI), mood (HADS), and coping (Brief COPE) between groups at week-12 using separate linear regression models controlling for the respective baseline values of the outcome variables. If necessary due to any imbalances between groups in baseline characteristics, we will control for selected demographic and clinical factors when examining the outcomes of interest. We will also test for potential moderators of the intervention effects using interaction terms for the linear models to assess whether differences in patient-reported outcomes are moderated by any clinical or socio-demographic factors (e.g., patient age, gender). Given the pilot nature of this study, we will use conservative (alpha = 0.05) and liberal (alpha = 0.15) values to assess statistical significance, especially as we evaluate outcomes in preparation for powering a subsequent full-scale efficacy trial. We will track and record reasons for all missing data (e.g., disease worsening, hospitalizations, hospice referral, lost to follow up, etc.). To address any missing data concerns, we will employ multiple imputation if data appear to be missing at random. If data are not missing at random, we will employ pattern mixture modeling.

Lastly, we will analyze the qualitative data with a multi-step process using coding and content analysis to explore patients’ perceptions and satisfaction regarding the mobile app content and features. This process will involve coding to structure data into categories and creating groups according to the broader issues or themes. We will identify major and minor themes within each content area, and we will extract and highlight messages. Two independent coders will examine discrepant, unexpected, or unclear data until agreement is reached. To assure the trustworthiness of our findings, we will take steps to maximize reliability and credibility including investigator triangulation (using a multidisciplinary team of investigators) and team debriefs of the interview content.

#### 2.4.1. Power analysis

The primary aim of the proposed study is to assess feasibility. We chose the sample size of 120 patients based on the feasibility of completing the project during the proposed timeframe and the ability to assess the preliminary efficacy of the intervention. The target sample size will provide us with preliminary data that can be used to determine within and between-group effect sizes and inform power analyses for future trials. Assuming 10% attrition within the sample of 120 participants, the study would have 80% power to detect between-group differences in mean change from baseline to 12 weeks of 0.54 standard deviation units (i.e., medium effect size) for the FACT-L and other secondary self-report outcomes.

## 3. Discussion

The goals of the present study are to develop and assess the feasibility, acceptability, and preliminary efficacy of a supportive care mobile app intervention for patients with advanced NSCLC. Prior work has demonstrated that these patients experience debilitating physical and emotional symptoms that are associated with poor QOL, and current guidelines recommend the early integration of palliative care with oncology care from the time of diagnosis and throughout the course of disease (Levy et al., 2016; Ferrell et al., 2017). However, the majority of patients with advanced lung cancer throughout the United States do not receive early palliative care in the ambulatory setting due to the lack of specialty trained clinicians and limited integration of palliative care in oncology (Hui et al., 2010; Lupu, 2010; Spetz et al., 2016; Lupu et al., 2018).

To enhance access to supportive care interventions aimed at improving QOL, our research team has begun to develop and test several mobile apps that patients can self-administer and learn strategies for enhancing symptom management and adaptive coping with serious illness (Fishbein et al., 2017; Greer et al., 2019, 2020). For example, in a recent trial, we tested the efficacy of a mobile app of a cognitive-behavioral therapy (CBT) intervention tailored to treat anxiety in sample of patients with diverse advanced cancers (Greer et al., 2019). Study patients reported significant improvements in anxiety symptoms and QOL, with the CBT app being most effective among the subgroup of patients with moderate to severe anxiety at baseline. These findings suggest that, despite limited supportive care resources that exist for patients, the use of novel technological interventions may provide one avenue for enhancing accessibility to evidence-based comprehensive care.

To our knowledge, the proposed study is the first that aims to examine the integration of a mobile app intervention into comprehensive care for patients with advanced lung cancer. Further, our app is the first of its kind to address and intervene upon multiple domains of quality of life that have not previously been targeted in patients with advanced cancer, such as social, functional, and existential wellbeing. The use of mobile app interventions, such as THRIVE, possess great promise for broad dissemination of evidence-based supportive and palliative care interventions in a timely, scalable, convenient, and potentially cost-effective manner. More specifically, mobile apps offer a useful approach for increasing accessibility for patients without creating an additional need for specialty-trained clinicians, which remains one of the major barriers to patients receiving such supportive care (Lupu, 2010; Lupu et al., 2018). Further, mobile apps can be implemented in a variety of care settings from large, academic medical centers to community oncology practices, reaching a wide array of patients with unmet needs. Their use may also reduce cost and staff resources dedicated to scheduling and providing in-person palliative and supportive care given the remote and asynchronous nature of the technology. Additionally, their integration may prove helpful for patients who face barriers to receiving in-person, clinic-based services given the degree of morbidity in the patient population or due to logistical challenges such as transportation.

It is important to note several limitations of the above protocol. First, only patients with advanced NSCLC are recruited to the study, and enrollment takes place at large academic cancer centers and their affiliates; thus, generalizability may be limited with respect to other disease groups or patients receiving care in other settings, such as stand-alone community oncology clinics. Further, only English-speaking patients are enrolled in the study, which ultimately may limit generalizability to patients of other racial or ethnic backgrounds. Additionally, the design of the present study precludes our ability to blind participants during study procedures or randomization. Also, despite efforts to maximize patient engagement with the intervention, it is possible that varying levels of technologic comfort between participants may affect the results.

Also of note, the follow-up assessment period is limited to 12 weeks after enrollment. As such, any longer-term effects of the intervention may become apparent after the trial period has ended, which would not be captured in the present study. In future trials and large-scale efficacy testing, we hope to evaluate the long-term intervention effects by utilizing a more prolonged follow-up period. Additionally, at this point, the app is solely designed to address patient needs despite prior research demonstrating that family and friend caregivers of patients also face physical and emotional challenges throughout the course of a loved one’s disease (Given et al., 2004; El-Jawahri et al., 2017). Future work is needed to develop a companion app to target caregiver concerns. Lastly, some participants may have supportive care needs that exceed the capacity of the app, which has important implications for future studies. Potential next steps include evaluation of a stepped model of care, such that participants who require services beyond that which is provided by the app are able to access traditional palliative care. This model may also more efficiently triage allocation of resources by reserving palliative care referral only for those with unremitting distress or an existing need not covered by the app.

Despite the aforementioned limitations, this project has the potential to impact all patients with advanced NSCLC, regardless of their location of care. A mobile app could be implemented across all oncology practice settings to ensure patients have access to care focused on helping them manage their symptoms and cope effectively with their illness. The primary aims of this study are to develop a supportive care mobile app that not only is acceptable to patients and feasible to implement, but also improves patients’ symptoms, coping, and QOL. As the first study of a mobile app focused on the core supportive care elements of the early palliative care model, the proposed intervention has the potential to be adapted further and benefit all patients with a serious cancer diagnosis.

## Ethics statement

The studies involving human participants were reviewed and approved by Dana-Farber/Harvard Cancer Center IRB. The patients/participants provided their written informed consent to participate in this study.

## Author contributions

JG, JT, AE-J, JJ, and LP contributed to the study conception and design. JG, JT, and AE-J wrote and developed the protocol. LW and JC wrote the first draft of the manuscript. JJ, LP, AE-J, JT, and JG contributed critically important revisions on previous versions of the manuscript. All authors contributed to the article and approved the submitted version.

## Funding

Funding for this study is provided by the National Comprehensive Cancer Network (NCCN), a non-profit organization, through a grant from AstraZeneca Pharmaceuticals, and is supported by CRP-20-097-01-PCSM from the American Cancer Society. NCCN and AstraZeneca Pharmaceuticals were not involved in the study design, collection, analysis, interpretation of data, the writing of this article or the decision to submit it for publication.

## Conflict of interest

JG has received compensation from BeiGene (advisor/consultant fees), Oxford University Press (royalties), and Springer (royalties). JG, JT, and AE-J also have received research funding from Blue Note Therapeutics.

The remaining authors declare that the research was conducted in the absence of any commercial or financial relationships that could be construed as a potential conflict of interest.

## Publisher’s note

All claims expressed in this article are solely those of the authors and do not necessarily represent those of their affiliated organizations, or those of the publisher, the editors and the reviewers. Any product that may be evaluated in this article, or claim that may be made by its manufacturer, is not guaranteed or endorsed by the publisher.

## References

[ref1] Andersson G. Cuijpers P. (2009). Internet-based and other computerized psychological treatments for adult depression: a meta-analysis. Cogn. Behav. Ther. 38, 196–205. doi: 10.1080/16506070903318960, PMID: 20183695

[ref2] Bangor A. Kortum P. T. Miller J. T. (2008). An empirical evaluation of the system usability scale. Int. J. Hum-Comput. Interact. 24, 574–594. doi: 10.1080/10447310802205776, PMID: 36882826

[ref3] Basch E. Deal A. M. Kris M. G. Scher H. I. Hudis C. A. Sabbatini P. . (2016). Symptom monitoring with patient-reported outcomes during routine Cancer treatment: a randomized controlled trial. J. Clin. Oncol. 34, 557–565. doi: 10.1200/JCO.2015.63.0830, PMID: 26644527 PMC4872028

[ref4] Bewick B. M. Trusler K. Barkham M. Hill A. J. Cahill J. Mulhern B. (2008). The effectiveness of web-based interventions designed to decrease alcohol consumption–a systematic review. Prev. Med. 47, 17–26. doi: 10.1016/j.ypmed.2008.01.005 18302970

[ref5] Bowen D. J. Kreuter M. Spring B. Cofta-Woerpel L. Linnan L. Weiner D. . (2009). How we design feasibility studies. Am. J. Prev. Med. 36, 452–457. doi: 10.1016/j.amepre.2009.02.002, PMID: 19362699 PMC2859314

[ref6] Carroll J. K. Moorhead A. Bond R. LeBlanc W. G. Petrella R. J. Fiscella K. (2017). Who uses Mobile phone health apps and does use matter? A secondary data analytics approach. J. Med. Internet Res. 19:e125. doi: 10.2196/jmir.5604, PMID: 28428170 PMC5415654

[ref7] Carver C. S. (1997). You want to measure coping but your protocol's too long: consider the brief COPE. Int. J. Behav. Med. 4, 92–100. doi: 10.1207/s15327558ijbm0401_6, PMID: 16250744

[ref8] Carver C. S. Scheier M. F. Weintraub J. K. (1989). Assessing coping strategies: a theoretically based approach. J. Pers. Soc. Psychol. 56, 267–283. doi: 10.1037/0022-3514.56.2.267, PMID: 2926629

[ref9] Cella D. F. Bonomi A. E. Lloyd S. R. Tulsky D. S. Kaplan E. Bonomi P. (1995). Reliability and validity of the functional assessment of Cancer therapy-lung (FACT-L) quality of life instrument. Lung Cancer 12, 199–220. doi: 10.1016/0169-5002(95)00450-F, PMID: 7655830

[ref10] Cella D. Eton D. T. Fairclough D. L. Bonomi P. Heyes A. E. Silberman C. . (2022). What is a clinically meaningful change on the functional assessment of Cancer therapy-lung (FACT-L) questionnaire? Results from eastern cooperative oncology group (ECOG) study 5592. J. Clin. Epidemiol. 55, 285–295. doi: 10.1016/S0895-4356(01)00477-2 11864800

[ref11] Cleeland C. S. Mendoza T. R. Wang X. S. Chou C. Harle M. T. Morrissey M. . (2000). Assessing symptom distress in cancer patients: the M.D. Anderson symptom inventory. Cancer 89, 1634–1646. doi: 10.1002/1097-0142(20001001)89:7<1634::AID-CNCR29>3.0.CO;2-V, PMID: 11013380

[ref12] Cuijpers P. Marks I. M. van Straten A. Cavanagh K. Gega L. Andersson G. (2009). Computer-aided psychotherapy for anxiety disorders: a meta-analytic review. Cogn. Behav. Ther. 38, 66–82. doi: 10.1080/16506070802694776, PMID: 20183688

[ref13] Cuijpers P. Turner E. H. Mohr D. C. Hofmann S. G. Andersson G. Berking M. . (2014). Comparison of psychotherapies for adult depression to pill placebo control groups: a meta-analysis. Psychol. Med. 44, 685–695. doi: 10.1017/S0033291713000457, PMID: 23552610

[ref14] Dionne-Odom J. N. Applebaum A. J. Ornstein K. A. Azuero A. Warren P. P. Taylor R. A. . (2018). Participation and interest in support services among family caregivers of older adults with cancer. Psychooncology 27, 969–976. doi: 10.1002/pon.4603, PMID: 29226997 PMC5840039

[ref15] El-Jawahri A. Greer J. A. Pirl W. F. Park E. R Jackson V. A Back A. L. . (2017). Effects of early integrated palliative care on caregivers of patients with lung and gastrointestinal Cancer: a randomized clinical trial. Oncologist 22, 1528–1534. doi: 10.1634/theoncologist.2017-0227, PMID: 28894017 PMC5728034

[ref16] Ferrell B. R. Temel J. S. Temin S. Alesi E. R. Balboni T. A. Basch E. M. . (2017). Integration of palliative care into standard oncology care: American Society of Clinical Oncology clinical practice guideline update. J. Clin. Oncol. 35, 96–112. doi: 10.1200/JCO.2016.70.1474, PMID: 28034065

[ref17] Fishbein J. N. Nisotel L. E. MacDonald J. J. Amoyal Pensak N. Jacobs J. M. Flanagan C. . (2017). Mobile application to promote adherence to Oral chemotherapy and symptom management: a protocol for design and development. JMIR Res Protoc 6:e62. doi: 10.2196/resprot.6198, PMID: 28428158 PMC5418526

[ref18] Given B. Wyatt G. Given C. Sherwood P. Gift A. DeVoss D. . (2004). Burden and depression among caregivers of patients with cancer at the end of life. Oncol. Nurs. Forum 31, 1105–1117. doi: 10.1188/04.ONF.1105-1117, PMID: 15547633 PMC1315286

[ref19] Greer J. A. Jacobs J. M. El-Jawahri A. Nipp R. D. Gallagher E. R. Pirl W. F. . (2018). Role of patient coping strategies in understanding the effects of early palliative care on quality of life and mood. J. Clin. Oncol. 36, 53–60. doi: 10.1200/JCO.2017.73.7221, PMID: 29140772 PMC5756320

[ref20] Greer J. A. Jacobs J. Pensak N. MacDonald J. J. Fuh C. X. Perez G. K. . (2019). Randomized trial of a tailored cognitive-behavioral therapy Mobile application for anxiety in patients with incurable Cancer. Oncologist 24, 1111–1120. doi: 10.1634/theoncologist.2018-0536, PMID: 30683710 PMC6693695

[ref21] Greer J. A. Jacobs J. M. Pensak N. Nisotel L. E. Fishbein J. N. MacDonald J. . (2020). Randomized trial of a smartphone Mobile app to improve symptoms and adherence to Oral therapy for Cancer. J. Natl. Compr. Cancer Netw. 18, 133–141. doi: 10.6004/jnccn.2019.7354, PMID: 32023526

[ref22] Greer J. A. Pirl W. F. Jackson V. A. Muzikansky A. Lennes I. T. Heist R. S. . (2012). Effect of early palliative care on chemotherapy use and end-of-life care in patients with metastatic non-small-cell lung cancer. J. Clin. Oncol. 30, 394–400. doi: 10.1200/JCO.2011.35.7996, PMID: 22203758

[ref23] Hagan T. L. Fishbein J. N. Nipp R. D. Jacobs J. M. Traeger L. Irwin K. E. . (2017). Coping in patients with incurable lung and gastrointestinal cancers: a validation study of the brief COPE. J. Pain Symptom Manag. 53, 131–138. doi: 10.1016/j.jpainsymman.2016.06.005, PMID: 27725249 PMC5191904

[ref24] Harris P. A. Taylor R. Thielke R. Payne J. Gonzalez N. Conde J. G. (2009). Research electronic data capture (REDCap)--a metadata-driven methodology and workflow process for providing translational research informatics support. J. Biomed. Inform. 42, 377–381. doi: 10.1016/j.jbi.2008.08.010, PMID: 18929686 PMC2700030

[ref25] Hoerger M. Greer J. A. Jackson V. A. Park E. R. Pirl W. F. (2018). Defining the elements of early palliative care that are associated with patient-reported outcomes and the delivery of end-of-life care. J. Clin. Oncol. 36, 1096–1102. doi: 10.1200/JCO.2017.75.6676, PMID: 29474102 PMC5891131

[ref26] Hui D. Elsayem A. De la Cruz M. Berger A. Zhukovsky D. S. Palla S. (2010). Availability and integration of palliative care at US cancer centers. JAMA 303, 1054–1061. doi: 10.1001/jama.2010.258, PMID: 20233823 PMC3426918

[ref27] Jacobsen J. Jackson V. Greer G. Temel J. What’s in the syringe? Principles of Early Integrated Palliative Care. Oxford: Oxford University Press, (2021).

[ref28] Levy M. Smith T. Alvarez-Perez A. Back A. Baker J. N. Beck A. C. . (2016). Palliative Care Version 1.2016. J. Natl. Compr. Cancer Netw. 14, 82–113. doi: 10.6004/jnccn.2016.0009, PMID: 26733557

[ref29] Lupu D. (2010). American Academy of H, palliative medicine workforce task F: estimate of current hospice and palliative medicine physician workforce shortage. J. Pain Symptom Manag. 40, 899–911. doi: 10.1016/j.jpainsymman.2010.07.004, PMID: 21145468

[ref30] Lupu D. Quigley L. Mehfoud N. Salsberg E. S. (2018). The growing demand for hospice and palliative medicine physicians: will the supply keep up? J. Pain Symptom Manag. 55, 1216–1223. doi: 10.1016/j.jpainsymman.2018.01.011, PMID: 29410071

[ref31] Mohr D. C. Burns M. N. Schueller S. M. Clarke G. Klinkman M. (2013). Behavioral intervention technologies: evidence review and recommendations for future research in mental health. Gen. Hosp. Psychiatry 35, 332–338. doi: 10.1016/j.genhosppsych.2013.03.008, PMID: 23664503 PMC3719158

[ref32] Morrison E. J. Novotny P. J. Sloan J. A. Yang P. Patten C. A. Ruddy K. J. . (2017). Emotional problems, quality of life, and symptom burden in patients with lung Cancer. Clin. Lung Cancer 18, 497–503. doi: 10.1016/j.cllc.2017.02.008, PMID: 28412094 PMC9062944

[ref33] Payne H. E. Lister C. West J. H. Bernhardt J. M. (2015). Behavioral functionality of mobile apps in health interventions: a systematic review of the literature. JMIR Mhealth Uhealth 3:e20. doi: 10.2196/mhealth.3335, PMID: 25803705 PMC4376122

[ref34] Ritterband L. M. Thorndike F. P. Gonder-Frederick L. A. Magee J. C. Bailey E. T. Saylor D. K. . (2009). Efficacy of an internet-based behavioral intervention for adults with insomnia. Arch. Gen. Psychiatry 66, 692–698. doi: 10.1001/archgenpsychiatry.2009.66, PMID: 19581560 PMC3723339

[ref35] Roeland E. J. Triplett D. P. Matsuno R. K. Boero I. J. Hwang L. Yeung H. N. . (2016). Patterns of palliative care consultation among elderly patients with Cancer. J. Natl. Compr. Cancer Netw. 14, 439–445. doi: 10.6004/jnccn.2016.0050, PMID: 27059192

[ref36] Siddiqi A. E. Sikorskii A. Given C. W. Given B. (2008). Early participant attrition from clinical trials: role of trial design and logistics. Clin. Trials 5, 328–335. doi: 10.1177/1740774508094406, PMID: 18697847 PMC2723836

[ref37] Spetz J. Dudley N. Trupin L. Rogers M. Meier D. E. Dumanovsky T. (2016). Few hospital palliative care programs meet National Staffing Recommendations. Health Aff (Millwood) 35, 1690–1697. doi: 10.1377/hlthaff.2016.0113, PMID: 27605652

[ref38] Steinhauser K. E. Clipp E. C. Hays J. C. Olsen M. Arnold R. Christakis N. A. . (2006). Identifying, recruiting, and retaining seriously-ill patients and their caregivers in longitudinal research. Palliat. Med. 20, 745–754. doi: 10.1177/0269216306073112, PMID: 17148529

[ref39] Sullivan D. R. Chan B. Lapidus J. A. Ganzini L. Hansen L. Carney P. A. . (1702). Association of Early Palliative Care use with Survival and Place of death among patients with advanced lung Cancer receiving Care in the Veterans Health Administration. JAMA Oncol. 2019:1709. doi: 10.1001/jamaoncol.2019.3105, PMID: 31536133 PMC6753505

[ref40] Temel J. S. Greer J. A. Admane S. Gallagher E. R. Jackson V. A. Lynch T. J. . (2011). Longitudinal perceptions of prognosis and goals of therapy in patients with metastatic non-small-cell lung cancer: results of a randomized study of early palliative care. J. Clin. Oncol. 29, 2319–2326. doi: 10.1200/JCO.2010.32.4459, PMID: 21555700

[ref41] Temel J. S. Greer J. A. El-Jawahri A. Pirl W. F. Park E. R. Jackson V. A. . (2017). Effects of early integrated palliative Care in Patients with Lung and GI Cancer: a randomized clinical trial. J. Clin. Oncol. 35, 834–841. doi: 10.1200/JCO.2016.70.5046, PMID: 28029308 PMC5455686

[ref42] Temel J. S. Greer J. A. Muzikansky A. Gallagher E. R. Admane S. Jackson V. A. . (2010). Early palliative care for patients with metastatic non-small-cell lung cancer. N. Engl. J. Med. 363, 733–742. doi: 10.1056/NEJMoa1000678, PMID: 20818875

[ref43] Vernon M. L. (2010). A review of computer-based alcohol problem services designed for the general public. J. Subst. Abus. Treat. 38, 203–211. doi: 10.1016/j.jsat.2009.11.001, PMID: 20015607 PMC2835799

[ref44] Zigmond A. S. Snaith R. P. (1983). The hospital anxiety and depression scale. Acta Psychiatr. Scand. 67, 361–370. doi: 10.1111/j.1600-0447.1983.tb09716.x, PMID: 6880820

